# Treatment of a Large Cohort of Childhood Chronic Noninfectious Uveitis in a Multicentric Large Study: Adalimumab Versus Methotrexate as First‐Line Therapy

**DOI:** 10.1002/art.70090

**Published:** 2026-03-12

**Authors:** Ilaria Maccora, Catherine Guly, Cinzia de Libero, Giulia Carreras, Athimalaipet V. Ramanan, Gabriele Simonini

**Affiliations:** ^1^ Pediatric Rheumatology Unit Meyer Children's Hospital IRCCS Florence Italy; ^2^ NeuroFARBA Department University of Florence Florence Italy; ^3^ Bristol Eye Hospital Bristol United Kingdom; ^4^ Pediatric Ophthalmology Unit Meyer Children's Hospital Florence Italy; ^5^ Biostatistic Unit Meyer Children's Hospital IRCCS Florence Italy; ^6^ Department of Paediatric Rheumatology Bristol Royal Hospital for Children Bristol United Kingdom; ^7^ Translational Health Sciences University of Bristol Bristol United Kingdom

## Abstract

**Objective:**

Treatment of childhood chronic idiopathic uveitis (cCIU) is predominantly based on studies in juvenile idiopathic arthritis–associated uveitis and expert opinion. Our aim was to report the treatment outcomes of our cohort of cCIU.

**Methods:**

Retrospective multicenter study involving the rheumatology and ophthalmology units at Florence, Italy, and Bristol, United Kingdom. We included children with cCIU, who received at least one systemic treatment. Ocular inflammation and treatment response were assessed according to the standardized uveitis nomenclature.

**Results:**

A total of 116 patients with cCIU received at least one systemic treatment (93 methotrexate, 22 adalimumab, and 1 mycophenolate), whereas 60 of them received an additional second‐line treatment (45 adalimumab, 14 mycophenolate, and 1 tocilizumab). Children treated with adalimumab (plus or minus methotrexate) as a first‐line therapy were more likely to achieve remission than methotrexate alone (χ^2^ = 31.35; *P* < 0.001); furthermore, children treated with methotrexate as a first‐line therapy relapsed earlier (χ^2^ = 4.35; *P* = 0.043). Children receiving adalimumab were more likely to stop treatment for remission than methotrexate (χ^2^ = 25.9; *P* < 0.001). Regarding second‐line therapy, children who started adalimumab (plus or minus methotrexate) were more likely to achieve remission than mycophenolate (χ^2^ = 14.66; *P* = 0.005). Children with nonanterior uveitis, conversely to the others, were more likely to achieve remission with adalimumab as a first‐line therapy than methotrexate (χ^2^ = 32.3; *P* < 0.001). Children with nonanterior uveitis, but not children with anterior uveitis, were more likely to stop the first‐line treatment when receiving adalimumab than methotrexate (χ^2^ = 18.56; *P* = 0.001) due to persistent remission.

**Conclusion:**

Adalimumab shows promise as a potential first‐line therapy for cCIU, particularly for posterior segment uveitis. Although effective in anterior uveitis, methotrexate leads children to relapse earlier than adalimumab.

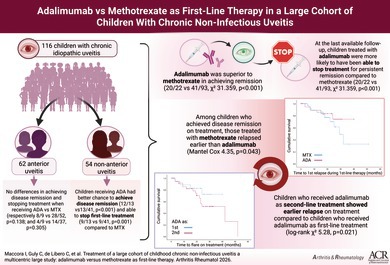

## Graphical abstract

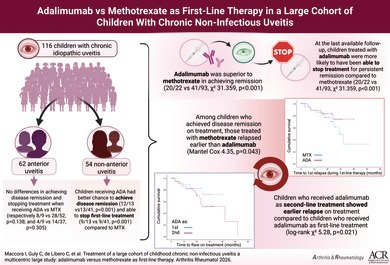

## INTRODUCTION

Juvenile idiopathic arthritis (JIA) is the most common cause of chronic uveitis in children, even though the approximately 30% to 40% of childhood uveitis^1^ has no known systemic cause and are termed childhood chronic idiopathic uveitis (cCIU) or undifferentiated uveitis. The Standardization of Uveitis Nomenclature (SUN) Working Group divides uveitis into different anatomic subtypes (anterior, intermediate, posterior, and panuveitis).^2^, ^3^, ^4^, ^5^

Timely treatment of cCIU is important to prevent ocular complications and blindness. Due to the sparse evidence on cCIU, treatment decisions are predominantly borrowed from studies on chronic uveitis in association with a systemic disease, such as JIA or Behçet disease, or from adult experience.^6^, ^7^, ^8^, ^9^

International recommendations advise use of topical and systemic glucocorticoids initially based on the severity and location of the uveitis, with the addition of a disease‐modifying antirheumatic drug (DMARD) or biologic agent, depending on the severity of disease, specific risk factors, persistent inflammation, or disease relapse.^6^, ^7^

Methotrexate is considered as the first‐line DMARD to be used. Adalimumab may be considered as a first‐line therapy in which there are severe ocular complications at baseline, although in the United Kingdom, adalimumab is only funded as a second‐line treatment for uveitis.^6^

Current treatment recommendations for childhood uveitis relate to JIA‐associated uveitis and anterior chronic idiopathic uveitis, and there is little guidance on the treatment of posterior segment uveitis in children.^6^, ^7^ The aim of our study was to report the therapeutic approach to a large cohort of cCIU, including all anatomic subtypes of uveitis.

## MATERIALS AND METHODS

### Study design

This is a retrospective, noninterventional, multicenter study involving cCIU diagnosed before 16 years of age. For the University Hospitals Bristol NHS Foundation Trust in Bristol (United Kingdom), data collection was approved by the hospital trust, and ethics committee approval was not required. Meyer Children's Hospital IRCCS ethics committee approved the study (27/2022) and informed consent was signed by legal guardian.

### Setting

This study involved the Rheumatology and Ophthalmology Units of the Meyer Children's Hospital IRCCS (Florence, Italy) and the Rheumatology and Ophthalmology Units of the University of Bristol and Weston NHS Foundation Trust in the United Kingdom.

### Study population

Children were included if they attended the rheumatology or the ophthalmology clinics of the two centers between January 1, 2019 and January 31, 2020, had a diagnosis of cCIU defined as persistent uveitis lasting more than three months with relapse less than months after discontinuing treatment according to SUN, no evidence of infection of other systemic disease, onset before 16 years old, have received at least one systemic treatment other than glucocorticoids before 18 years of age, and have a follow‐up of at least three months after starting the systemic treatment. Patients were excluded from this study if they had a diagnosis of a systemic disease, previous malignancy, demyelinating disease, or cerebral vasculitis.

### Data collection

Data were retrospectively collected from medical records at the onset of the disease, at the start of each systemic therapy, at 3 months (±2 weeks), 6 months (±2 weeks), 12 months (±2 weeks), 2 years (±1 month), 3 years (±1 month), and after each systemic treatment change. The baseline was recorded as the first presentation of disease, which in some cases was not in the hospital follow‐up.

The following data were collected: demographics including age, biologic sex at birth, ethnicity, age at onset, duration of disease, and age at diagnosis (calculated considering the first visit when the diagnosis of uveitis was made). When the precise date was not available, and the month and year of onset were the only data accessible, the 15th of the month was considered as onset. Laboratory data included antinuclear antibody (ANA) status, and parameters of inflammation at onset if available (C‐reactive protein [normal value <0.5 mg/dl], and erythrocyte sedimentation rate [ESR] [normal value <10 mm/h]) were collected.

Clinical characteristics of uveitis included laterality of uveitis (unilateral or bilateral), anatomic location according to SUN^2^ as anterior, intermediate, anterior and intermediate, posterior, and panuveitis. Symptoms at onset were recorded as pain, redness floaters, or blurred vision.

Ocular complications were recorded at onset and at the last available follow‐up as present or absent for each eye. The following complications were recorded: cataract, elevated intraocular pressure (>21 mm Hg), ocular hypotony (<5 mm Hg), optic disc swelling, macular edema on optical coherence tomography scan, posterior synechiae, epiretinal membrane, band keratopathy, and choroidal neovascular membrane.

Best corrected visual acuity (VA) was recorded in Logarithm of the Minimum Angle of Resolution (LogMAR), and when this scale was not available, the appropriate conversion was performed according to Schulze‐Bonsel et al.^10^ VA has been stratified as <0.4 LogMAR, ≥0.4 and <1 LogMAR, and blindness if ≥1 LogMAR.

Ocular inflammatory activity in the anterior chamber was graded using anterior chamber cells on slit‐lamp examination according to SUN guidelines. Vitreous haze was assessed by binocular indirect ophthalmoscopy score or National Eye Institute vitreous haze scale.^2^, ^5^ Evaluation of retinal or choroidal lesions on slit‐lamp examination, presence of active vasculitis on fluorescein angiography (where performed), and presence or absence of cystoid macular edema on optical coherence tomography were recorded. Drug treatments, concomitant therapy, time to treatment effect, duration of treatment, response to treatment, cessation of therapy, and uveitis flares during and after treatment were also recorded.

The response to treatment was assessed according to SUN criteria as follows:

1. Response was defined as the improvement of intraocular inflammation considered as anterior chamber cells, according to the definition of improvement of the SUN Working Group criteria for anterior uveitis.^2^ Anterior chamber inflammation was considered “inactive” or controlled if the inflammatory activity was grade 0 cells. Regarding posterior inflammation, the National Eye Institute system for grading vitreous haze was adopted, and the designation “trace” was recorded as 0.5+. Uveitis was defined as improved and treatment as successful when its activity decreased by two steps in the level of inflammation (anterior chamber cells and/or vitreous haze) with two drops or less per day or decreased to grade 0 at three‐months and six‐months follow‐up (±2) and no declaration of treatment failure due to intolerability or safety concerns at three and six months.
2. Remission on treatment: recorded as present or absent. Remission on treatment is defined as anterior chamber cells ≤0.5, absence of optic disc edema, absence of macular edema, vitreous haze ≤0.5 plus absence of active vasculitis, and the absence of topical and systemic glucocorticoids for three consecutive months.
3. Time to achieve inactive disease, defined as above reported, is counted as months after starting systemic treatment.

The main measures of outcomes considered were the achievement of remission on treatment, the time to achieve inactivity, flare on treatment, the ability to stop the treatment, and time to flare after the withdrawal of treatment.

### Statistical analysis

Statistical analysis was performed using SPSS version 29.0 for Windows and STATA version 15 (StataCorp LLC, College Station, TX). Continuous variables were described using median and interquartile range (IQR). For dichotomous and/or categorical variables, proportions were used to assess the clinical characteristic of the population. Statistical significance (*P* value) was assessed at the 0.05 level. Chi‐square test and Fisher test were used to compare categorical variables as appropriate, whereas continuous variables were compared using nonparametric tests.

The association between achievement of remission on therapy, relapse on therapy, drug discontinuation due to persistent remission and relapse after drug withdrawal with the therapy, and a selected set of variables was assessed using multivariable logistic regression models. To describe the time to event outcomes (eg, time to remission, time to relapse on treatment), Kaplan‐Meier survival curves were generated, and log‐rank tests were used to compare survival functions across treatments. The association of these outcomes with treatment and a set of selected variables was assessed using Cox proportional hazards models. Predictors were selected based on clinical hypotheses and included, in addition to therapy, sex, age at onset, disease duration, ANA positivity, ocular complications at presentation, and baseline LogMAR.

Due to the substantial proportion of missing values observed, we employed multiple imputation by chained equations, performing 50 imputations. Only covariates (predictor variables) were imputed; outcomes were left untouched, to preserve the integrity of the observed event data. The number of imputations (50) was chosen because some covariates had missingness rates as high as 30% to 40%, and increasing the number of imputations helps to reduce Monte Carlo error and ensure stable and reliable pooled estimates in cases of high missing data proportions. Multivariate models were then fitted to each of the 50 imputed datasets, and pooled estimates—including coefficients, standard errors, and confidence intervals (CIs)—were derived using Rubin's rules. This strategy ensures that the imputation process does not artificially alter observed outcome distributions, reducing bias in parameter estimation while increasing statistical efficiency despite missing predictor data.

### Data statement

Data will be made available on reasonable request to the corresponding author.

## RESULTS

There were 146 patients with cCIU (76 boys and 42 ANA positive), of whom 116 met the inclusion criteria for the study (79.4%) (Supplementary Figure 1). All patients had received at least one systemic treatment other than glucocorticoids, and 60 had received a second‐line treatment (41.1%) with a median follow‐up of 52 months (IQR 35–79). The characteristics of the 116 patients included in the study are summarized in Table 1. We observed that children with panuveitis have a significantly higher ESR (*P* = 0.0019), present a higher number of ocular complications at the onset of the disease (*P* = 0.0131), and have lower VA (*P* = 0.0192) compared with other anatomic subtypes of uveitis (see Table 1). At the onset of the disease, children with posterior segment uveitis were more likely to have impaired VA (<0.4 LogMAR 23 of 33 vs 0.4–1 LogMAR 5 of 15; blindness 2 of 8; χ^2^ = 8.54; *P* = 0.014), even when expressed as continuous variables (0.413 vs 0.023; *P* = 0.001).

**Table 1:** Ocular and laboratory characteristics of the population included*

Variable	Whole cohort (n = 116)	Anterior (n = 62; 53.9%)	Intermediate (n = 25; 20.9%)	ant+intermediate (n = 13; 11.3%)	Panuveitis (n = 16; 13.9%)	*P* value^a^
Female sex, n (%)	55 (47.4)	29 (46.8)	11 (44.0)	6 (46.2)	9 (56.3)	0.888
Age at onset, median (IQR)	110 (75–134)	98 (68–136)	118 (105–133)	105 (75–113)	114 (94–132)	0.2998
Duration, median (IQR)	52 (35–79)	47 (33–68)	70 (48–92)	55 (31–60)	59 (42–81)	0.1093
ANA positivity,^b^ n (%)	33 (28.5)	23 (37.1)	4 (16.0)	1 (7.8)	5 (31.3)	0.256^c^
ESR,^d^ median (IQR), mm/h	10 (2–23)	16 (7–24)	2 (2–9)	9 (6–19)	29 (23–38)	0.0019
CRP,^e^ median (IQR), mg/dl	0.1 (0.1–0.3)	0.1 (0.1–0.3)	0.3 (0.1–0.4)	0.2 (0.1–0.6)	0.1 (0.1–0.3)	0.2794
Bilateral uveitis, n (%)	98 (84.5)	51 (82.3)	21 (84.0)	12 (92.3)	14 (87.5)	0.812
Symptomatic uveitis,^f^ n (%)	88 (77.9)	43 (70.5)	19 (79.2)	12 (92.3)	14 (93.3)	0.132^g^
Children with complications at onset,^h^ n (%)	80 (69.0)	43 (69.4)	15 (60.0)	10 (76.9)	12 (75.0)	0.467
Complications,^h^ n (%)	2 (1–4)	2 (1–4)	1 (0–2)	2 (1–4)	2 (2–4)	0.0131
BCVA onset						0.0192
LogMAR,^i^ median (IQR)	0.2 (0.0–0.5)	0 (0.0–0.3)	0.4 (0.2–0.8)	0.3 (0.0–0.4)	0.3 (0.0–0.6)	
<0.3, n (%)	49 (42.2)	27 (43.6)	7 (28.0)	8 (61.5)	7 (43.8)	
0.4–1, n (%)	21 (18.1)	6 (9.7)	8 (32.0)	3 (23.1)	4 (25.0)	0.242
>1, n (%)	11 (9.5)	3 (4.8)	4 (16.0)	2 (15.4)	2 (12.5)	

* ANA, antinuclear antibody; ant+intermediate, anterior and intermediate; BCVA, best corrected visual acuity; CRP, C‐reactive protein; ESR, erythrocyte sedimentation rate; IQR, interquartile range; LogMAR, Logarithm of the Minimum Angle of Resolution.

^a^ *P* values are computed from chi‐square test for categorical variables and from Kruskal‐Wallis test for continuous variables.

^b^ Completed in 92 patients.

^c^ χ^2^ = 7.7653.

^d^ Completed in 43 patients.

^e^ Completed in 68 patients.

^f^ Completed in 113 patients.

^g^ χ^2^ = 5.605.

^h^ Completed in 98 patients.

^i^ Completed in 81 patients.

The median time for the administration of the first systemic treatment was six months (IQR 2–9). Methotrexate was prescribed as the first‐line immunosuppressive treatment in 93 children (80.2%), 22 received adalimumab (19%), of whom 5 had concomitant methotrexate, and 1 child was given mycophenolate mofetil (MMF) (0.9%). For second‐line treatment, 45 children received adalimumab (75%), 14 received MMF (23.3%), and 1 received tocilizumab (1.7%) (Table 2). At the last available follow‐up, we observed that there was a significant improvement in the best corrected VA expressed in LogMAR (*P* < 0.001) and stratified (*P* < 0.001) in the number of children with complications (*P* = 0.011) and in the number of complications (*P* < 0.001) (Supplementary Table 1).

**Table 2:** Treatment performed in a cohort of childhood chronic noninfectious uveitis*

Treatment	First course of systemic treatment					Second course of systemic treatment				
	Overall (n = 116)	MTX (n = 93; 80.2%)	MMF (n = 1, 0.9%)	ADA (n = 22, 19.0%)	*P* value	Overall (n = 60)	ADA (n = 45, 75%)	MMF (n = 14, 23.3%)	TCZ (n = 1, 1.7%)	*P* value
Concomitant therapy	5 MTX	–	–	5 MTX	–	40 MTX, 1 MMF	39 MTX, 1 MMF	–	1 MTX	–
Time of administration,^a^ median (IQR)	6 (2–9)	5 (2–9)	4 (4–4)	7 (4–10)	0.1726	13 (7–24)	12 (7–19)	16 (8–31)	21 (21–21)	0.3211
Duration of therapy,^b^ median (IQR)	25 (14–39)	24 (11–40)	5 (5–5)	33 (24–39)	0.1141	31 (12–41)	27 (15–40)	34 (7–49)	–	0.5011
Achievement of remission on therapy,^c^ n (%)	61 (52.6)	41 (44.1)	–	20 (90.1)	<0.001^d^	49 (81.7)	40 (88.9)	9 (64.3)	–	0.005^e^
Time to achieve remission,^f^ median (IQR)	6 (5–7)	6 (5–6.5)	–	6 (5–7.5)	0.9796	6 (5–9)	6 (5–9)	9 (5–11)	–	0.4195
Relapse on therapy in patients who achieved remission,^g^ n (%)	16 (26.2)	12 (29.3)	–	4 (20.0)	0.440^h^	21 (42.9)	17 (42.5)	4 (44.4)	–	0.891^i^
Time to first relapse on treatment in patients who achieved remission,^j^ median (IQR)	18 (10–25)	17 (10–23)	–	23 (16–28)	0.1555	18 (9–25)	18 (9–27)	18 (12–21)	–	0.7048
Pts stopping drug for persistent remission,^k^ n (%)	36 (31.0)	23 (24.7)	–	13 (59.1)	<0.001^l^	26 (43.3)	22 (48.9)	4 (28.6)	–	0.077^m^
Uveitis relapse after stopping drug,^n^ n (%)	20 (55.6)	12 (52.2)	–	8 (61.5)	0.587^o^	19 (76.0)	18 (81.8)	1 (33.3)	–	0.065^p^
Time free from relapse after stopping drug,^q^ median (IQR)	5 (3–19)	6 (2–21)	–	4 (4–17)	0.6791	6 (5–12)	6 (5–12)	7 (4–25)	–	0.8815

* ADA, adalimumab; MMF, mycophenolate mofetil; MTX, methotrexate; IQR, interquartile range; TCZ, tocilizumab.

^a^ n = 116 in first course; n = 60 in second course.

^b^ n = 114 in first course; n = 58 in second course.

^c^ n = 115 in first course; n = 59 in second course.

^d^ χ^2^ = 31.3591.

^e^ χ^2^ = 14.6662.

^f^ n = 59 with achievement of remission in first course; n = 47 in second course.

^g^ n = 61 in first course; n = 49 in second course.

^h^ χ^2^ = 0.5968.

^i^ χ^2^ = 0.2319.

^j^ n = 61 in first course; n = 48 in second course.

^k^ n = 115 in first course; n = 60 in second course.

^l^ χ^2^ = 25.9040.

^m^ χ^2^ = 8.4322.

^n^ n = 36 in first course; n = 26 in second course.

^o^ χ^2^ = 0.2950.

^p^ χ^2^ = 3.4024.

^q^ n = 36 in second course; n = 25 in second course.

**Figure 1:**
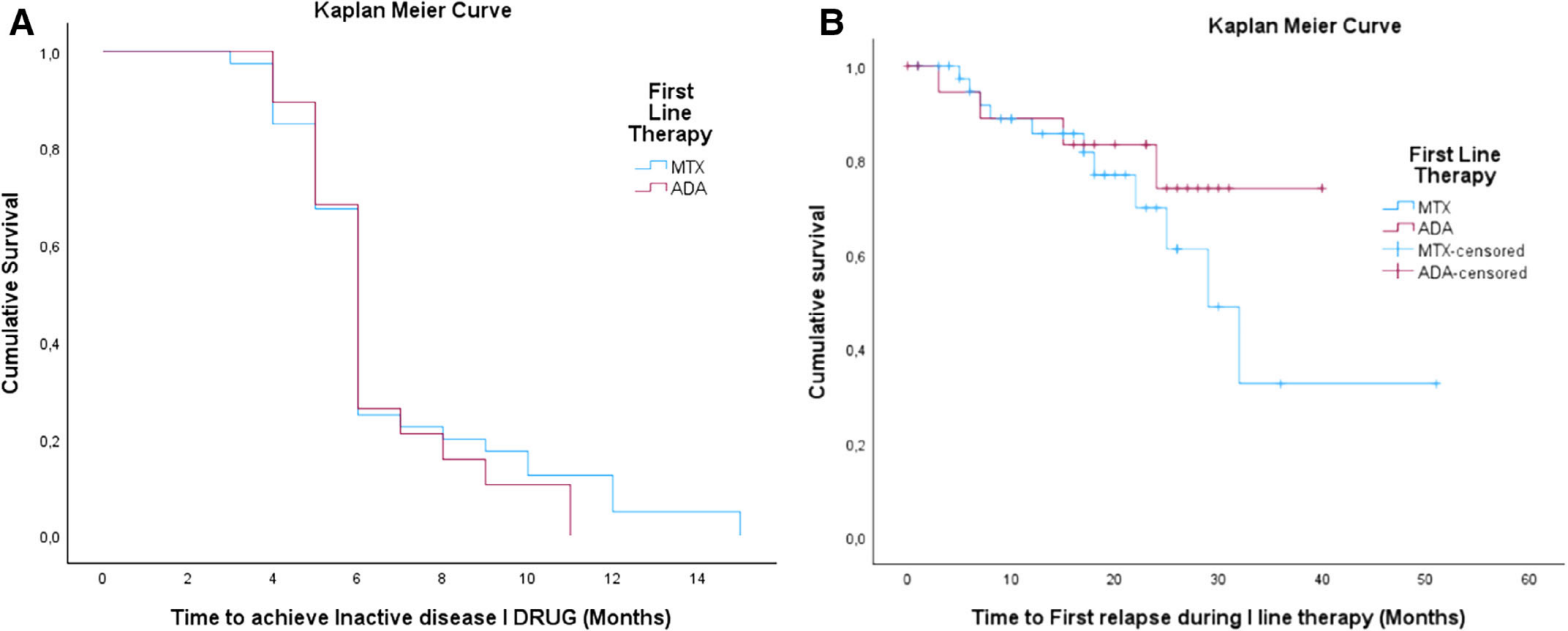
(A) Survival curve of the time to achieve remission on treatment with the first‐line treatment (log‐rank, χ^2^ = 0.41; *P* = 0.522). (B) Survival curve of the time to the first relapse on treatment after achieving remission with first‐line treatment (log‐rank, χ^2^ = 4.35; *P* = 0.043). ADA, adalimumab; MTX, methotrexate.

The 22 patients, 5 of them with concomitant methotrexate, receiving adalimumab as first‐line therapy were more likely to achieve ocular remission than those on methotrexate alone (20 of 22 vs 41 of 93; χ^2^ = 31.359; *P* < 0.001). No difference in time to achieve remission on treatment (Kruskal‐Wallis *P* = 0.9796 on those who reached remission; log‐rank χ^2^ = 0.41; *P* = 0.519; Figure 1A) has been detected between these two groups. The child who received MMF as first‐line therapy did not achieve disease remission with this treatment. Among children who achieved remission, there were no significant differences in frequency of relapse on therapy (12 of 41 vs 4 of 20; χ^2^ = 0.597; *P* = 0.440), but children receiving methotrexate alone relapsed significantly earlier compared with children on adalimumab (median 17 vs 23 months; Kruskal‐Wallis *P* = 0.155; Mantel Cox χ^2^ = 4.35, *P* = 0.043, corrected for the duration of treatment; odds ratio [OR] 1.027; CI 1.001–1.0054; Figure 1B).

At the last available follow‐up, children who were on adalimumab as first‐line treatment (plus or minus methotrexate) were significantly more likely to have stopped the treatment for persistent remission than methotrexate alone (13 of 22 vs 23 of 93; χ^2^ = 25.90; *P* < 0.001) (Table 2). The number of relapses and the time to relapse after drug withdrawal were not significantly different between the two groups (Table 2).

**Figure 2:**
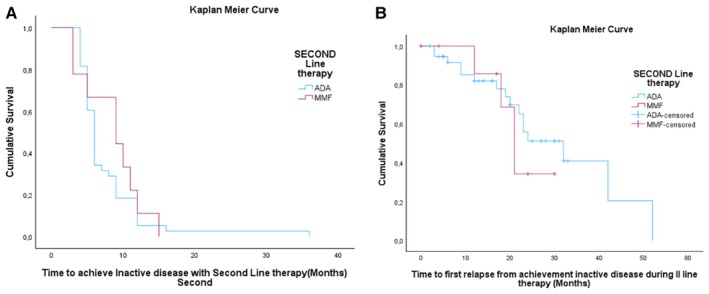
(A) Survival curve of the time to achieve remission on treatment with the second‐line treatment (log‐rank, χ^2^ = 0.294; *P* = 0.587). (B) Survival curve of the time to the first relapse on treatment after achieving remission with second‐line treatment (log‐rank, χ^2^ = 2.90; *P* = 0.100). ADA, adalimumab; MMF, mycophenolate mofetil. Color figure can be viewed in the online issue, which is available at http://onlinelibrary.wiley.com/doi/10.1002/art.70090/abstract.

Addressing second‐line therapy, adalimumab was the most frequently administered drug given to 45 of 60 children (75%), in 39 with concomitant methotrexate and in 1 with mycophenolate, followed by mycophenolate administered to 14 of 60 children (23%) (Table 2). Children who received adalimumab (plus or minus methotrexate) as a second‐line therapy were more likely to achieve remission on treatment compared with mycophenolate (40 of 45 vs 9 of 14; χ^2^ = 14.6662; *P* = 0.005), with no difference in time to achieve disease remission (Table 2; Figure 2A). We did not observe a significant difference in relapse on treatment (Table 2), time to relapse on treatment (Figure 2B), number of patients who stopped treatment, relapse after drug withdrawal, and time to relapse after drug withdrawal between the two groups. Additionally, we stratified the analyses of outcomes of the 116 patients with cCIU who received the first systemic therapy based on the different nature of anatomic subtype of uveitis and clustering by anterior involvement (61; 52%) versus posterior segment uveitis (55; 47%).

Children with posterior segment uveitis were more likely to achieve disease remission when receiving adalimumab (plus or minus methotrexate) as the first‐line treatment compared with children who received methotrexate (12 of 13 vs 13 of 41; χ^2^ = 32.3024; *P* < 0.001); conversely, children with anterior uveitis did not show significant differences in disease remission when receiving adalimumab (plus or minus methotrexate) or methotrexate as first‐line treatment (8 of 9 vs 28 of 52; χ^2^ = 3.9558; *P* = 0.138). Similarly, children with posterior segment uveitis were more likely to stop the first‐line treatment for persistent remission when receiving adalimumab (plus or minus methotrexate) compared with methotrexate (9 of 13 vs 9 of 41; χ^2^ = 18.5608; *P* = 0.001); conversely, children with anterior uveitis did not show significant differences in treatment withdrawal for persistent remission when receiving adalimumab (plus or minus methotrexate) or methotrexate as first‐line treatment (4 of 9 vs 14 of 37; χ^2^ = 1.05; *P* = 0.305).

In patients receiving methotrexate, the following factors did not affect the chance of achieving remission: biologic sex, ANA positivity, ESR at onset, the number of complications, and the type of complications at the disease onset. However, children with lower VA at onset were less likely to achieve disease remission with methotrexate (0.413 vs 0.023; *P* = 0.001).

Additionally, treatment outcomes of patients receiving adalimumab (plus or minus methotrexate) as first‐line immunosuppressive treatment were compared with patients receiving adalimumab (plus or minus methotrexate) after conventional DMARD failure. No significant differences were found between the two groups in ocular remission on treatment (20 of 22 vs 40 of 45), relapse on treatment (4 of 22 vs 16 of 39), stopping treatment due to persistent remission (12 of 22 vs 22 of 40), relapse after drug withdrawal (7 of 12 vs 18 of 22), time to achieve remission on treatment (median 6 vs 6 months), and time to flare after drug withdrawal for persistent remission (median 4 vs 6.5 months). However, children who received adalimumab (plus or minus methotrexate) as second‐line treatment showed earlier relapse on treatment compared with children who received adalimumab (plus or minus methotrexate) as first‐line treatment (log‐rank χ^2^ = 5.28; *P* = 0.021; Supplementary Figure 2).

Results from the multivariate logistic regression models applied to the imputed datasets (Table 3) indicate that children treated with adalimumab had significantly higher odds of achieving disease remission compared with those treated with methotrexate alone (OR 17.87; 95% CI 3.44–92.72), and children with nonanterior uveitis had significantly lower odds of achieving disease remission (OR 0.33; 95% CI 0.12–0.88). After adjusting for covariates, children receiving adalimumab are approximately 18 times more likely to achieve remission, whereas children with nonanterior uveitis are 0.3 times less likely to achieve disease remission. Moreover, the duration of therapy was significantly associated with relapse on therapy (OR 1.12; 95% CI 1.03–1.22) and with stopping drug for persistent remission (OR 0.91; 95% CI 0.85–0.97), whereas disease duration was significantly associated with stopping drug for persistent remission (OR 1.09; 95% CI 1.04–1.15) and nonanterior uveitis with stopping the drug (OR 7.51; 95 CI 1.24–45.4). The Cox proportional hazards models for time to achieve remission, time to relapse on treatment, and time to relapse out of therapy showed a significant association between relapse on treatment and duration of therapy (OR 1.04; 95% CI 1.00–1.08) (Table 4).

**Table 3:** Pooled results from the multivariable logistic regression models for achievement of remission, relapse on therapy, and stopping drug for persistent remission (among patients who achieved remission) and relapse out of therapy (among patients who stop drug) after multiple imputation (50 datasets)*

Variable	Achievement of remission	Relapse on therapy	Stop drug for persistent remission	Relapse out of therapy
Treatment, OR (95% CI)				
MTX	1	1	1	1
ADA	**17.87 (3.44–92.72)**	0.39 (0.07–2.26)	0.75 (0.14–4.14)	2.58 (0.34–19.68)
Duration of therapy, OR (95% CI)	1.00 (0.98–1.03)	**1.12 (1.03–1.22)**	**0.91 (0.85–0.97)**	1.01 (0.94–1.09)
Sex, OR (95% CI)				
Boys	1	1	1	1
Girls	0.67 (0.27–1.68)	1.51 (0.32–7.09)	0.22 (0.04–1.12)	0.39 (0.05–2.92)
Age, OR (95% CI)	1.01 (0.99–1.02)	1.00 (0.97–1.02)	1.01 (0.99–1.03)	0.97 (0.93–1.01)
Disease duration, OR (95% CI)	1.00 (0.98–1.02)	1.00 (0.97–1.03)	**1.09 (1.04–1.15)**	1.03 (0.98–1.08)
Anatomic location, OR (95% CI)				
Anterior	1	1	1	1
Nonanterior	**0.33 (0.12–0.88)**	0.42 (0.08–2.30)	**7.51 (1.24–45.4)**	0.73 (0.09–5.73)
ANA positivity, OR (95% CI)				
Yes	1	1	1	1
No	0.84 (0.25–2.79)	1.56 (0.23–10.77)	1.09 (0.16–7.21)	0.59 (0.06–5.88)
Complications, OR (95% CI)				
Yes	1	1	1	1
No	0.93 (0.25–3.48)	0.83 (0.08–8.87)	0.67 (0.09–4.95)	5.6 (0.63–49.48)
LogMAR onset, OR (95% CI)	0.67 (0.22–2.10)	0.97 (0.22–4.30)	0.59 (0.12–2.95)	1.06 (0.07–16.28)

* Bold indicates significant values. ADA, adalimumab; ANA, antinuclear antibody; CI, confidence interval; LogMAR, Logarithm of the Minimum Angle of Resolution; MTX, methotrexate; OR, odds ratio.

**Table 4:** Pooled results from the multivariable Cox proportional hazard regression model for time to achievement of remission, relapse on therapy, and relapse out of therapy after multiple imputation (50 datasets)*

Variable	Achievement of remission with first‐line treatment	Relapse on treatment after remission with first line	Relapse out of therapy
Treatment, HR (95% CI)			
MTX	1	1	1
ADA	1.29 (0.70–2.36)	0.69 (0.18–2.67)	1.00 (0.34–2.92)
Duration of therapy, HR (95% CI)	0.99 (0.97–1.02)	**1.04 (1.00–1.08)**	1.02 (0.96–1.08)
Sex, HR (95% CI)			
Boys	1	1	1
Girls	1.24 (0.70–2.21)	1.72 (0.52–5.71)	0.54 (0.16–1.86)
Age, HR (95% CI)	1.00 (0.99–1.01)	1.00 (0.98–1.02)	0.98 (0.95–1.01)
Disease duration, HR (95% CI)	1.00 (0.99–1.01)	0.99 (0.97–1.01)	1.00 (0.98–1.03)
Anatomic location, HR (95% CI)			
Anterior	1	1	1
Nonanterior	0.80 (0.45–1.42)	0.43 (0.10–1.88)	1.20 (0.42–3.4)
ANA positivity, HR (95% CI)			
Yes	1	1	1
No	0.90 (0.44–1.82)	1.27 (0.31–5.27)	0.62 (0.14–2.85)
Complications, HR (95% CI)			
Yes	1	1	1
No	0.61 (0.29–1.32)	0.73 (0.13–4.07)	2.89 (0.64–13.1)
LogMAR onset, HR (95% CI)	0.85 (0.50–1.43)	1.35 (0.41–4.51)	0.68 (0.18–2.6)

* Bold indicates significant values. ADA, adalimumab; ANA, antinuclear antibody; CI, confidence interval; HR, hazard ratio; LogMAR, Logarithm of the Minimum Angle of Resolution; MTX, methotrexate.

## DISCUSSION

To the best of our knowledge, this is one of the largest cohorts reporting the therapeutic outcomes and course of cCIU in children. Adalimumab plus or minus methotrexate, a key drug for JIA‐associated uveitis,^11^, ^12^ outperformed methotrexate as first‐line treatment in achieving ocular remission in our cohort of children with idiopathic uveitis, particularly in posterior segment uveitis. Conversely, in anterior uveitis, there was no significant difference in disease remission between methotrexate and adalimumab, and methotrexate can be still considered as a valid treatment option. However, of those who achieved remission with first‐line therapy, children who were treated with methotrexate alone relapsed more quickly than children treated with adalimumab. Additionally, a higher proportion of children on adalimumab were able to discontinue treatment due to persistent remission. These results echo clinical trial data in JIA‐associated uveitis of Ramanan et al and Quartier et al, who demonstrated that adding adalimumab to methotrexate significantly suppressed uveitis activity, reduced the requirement for topical steroids, and delayed treatment failure compared with placebo.^11^, ^12^

Furthermore, the beneficial effects of adalimumab on childhood uveitis demonstrated in our cohort are in line with previous case series and cohort studies with different subtypes of uveitis, including JIA‐associated uveitis. They showed efficacy of adalimumab for childhood uveitis but did not compare it to methotrexate effect.^13^, ^14^, ^15^, ^16^, ^17^, ^18^, ^19^, ^20^, ^21^ Indeed, in our cohort, methotrexate was still the most common first‐line treatment used for the management of pediatric noninfectious uveitis, in keeping with national and international guidelines and the published literature.^6^, ^7^, ^8^, ^9^, ^15^, ^19^, ^20^, ^22^, ^23^, ^24^ However, methotrexate achieved uveitis remission in only 44% of our cases; this is in contrast with other studies reporting uveitis remission in around 73% of cases, possibly reflecting the more refractory nature of our cohort that included posterior segment as well as anterior uveitis.^24^ When looking at the subcohorts, adalimumab (plus or minus methotrexate) appeared to show a significant benefit over methotrexate in posterior segment uveitis compared with anterior uveitis, but the number on adalimumab in the anterior uveitis group was smaller, which may have impacted the results.

Moreover, children in our cohort treated with methotrexate showed a significant earlier relapse of uveitis compared with those on adalimumab, which aligns with the work of Tirelli et al,^25^ who showed that patients with JIA‐associated uveitis have a low chance of maintaining long‐lasting uveitis remission on methotrexate. Children treated with adalimumab as first‐line treatment have a greater chance of discontinuing the therapy than those treated with methotrexate alone, which is likely to be due to better disease control with adalimumab.

Adalimumab is recommended as a second‐line immunosuppressive agent for children with uveitis, including idiopathic uveitis.^4^, ^6^, ^7^, ^8^, ^11^, ^12^, ^16^, ^17^, ^22^, ^26^, ^27^, ^28^ In our cohort, comparing adalimumab used as a first‐line versus using it as second‐line agent, the ability to achieve remission and the proportion of patients who relapsed were similar. However, when adalimumab was used as a second‐line agent, it was associated with an earlier relapse on therapy compared with use as a first‐line therapy. Better performance of adalimumab when used as a first‐line treatment has also been previously reported, although in a smaller and heterogenous cohort of 26 children with different subtypes of uveitis.^17^

There are several limitations with the study. The retrospective nature of the study, as well as the characteristics of the two recruiting centers, both of which are tertiary centers, will have introduced selection and referral bias. Some of the subcohorts of patients had small numbers of patients, hampering the chance to perform additional comparisons (ie, comparing the performance of adalimumab alone versus adalimumab with methotrexate). Adalimumab is only funded as a second‐line immunosuppressive therapy in the United Kingdom; thus, adalimumab as a first‐line approach mostly comes from the Florence, Italy center. Missing data and limited length of follow‐up after treatment withdrawal will have affected the data analysis. Data on adalimumab failure secondary to antibody development were not available. Partial response was not assessed due to the limited number of patients.

Drug doses and subtherapeutic or tapering doses of drugs were not recorded. Multiple imputation was used to take into account for missing data uncertainty, leading to more accurate standard errors and unbiased results. However, this methodology involves increased complexity, computation time, and potential bias if imputation models are mis‐specified, requiring careful implementation. It is superior to single imputation for robust inferences but demands more resources than simpler methods.

Treatment of cCIU is challenging with disease remission using the first‐line treatment achieved in only 50% of patients. Adalimumab plus or minus methotrexate shows promise as a first‐line treatment for cCIU, and it is possible that patients with posterior segment uveitis, who tend to have worse VA at presentation, would benefit from more aggressive treatment with biologics from the disease onset. Prospective, randomized trials comparing methotrexate and adalimumab as an initial systemic therapy in pediatric uveitis, in both anterior and posterior segment cCIU, would help define optimal treatment algorithms. Longevity of cCIU remission with adalimumab monotherapy also deserves further study, including the risk of drug antibody formation. Effective treatment of cCIU is important not only for reducing ocular damage and improving visual outcomes for children but also for managing direct and indirect health care costs.

## AUTHOR CONTRIBUTIONS

All authors contributed to at least one of the following manuscript preparation roles: conceptualization AND/OR methodology, software, investigation, formal analysis, data curation, visualization, and validation AND drafting or reviewing/editing the final draft. As corresponding author, Dr Maccora confirms that all authors have provided the final approval of the version to be published and takes responsibility for the affirmations regarding article submission (eg, not under consideration by another journal), the integrity of the data presented, and the statements regarding compliance with institutional review board/Declaration of Helsinki requirements.

## Supporting information


**Disclosure Form**:

**Data S1.** Supporting Information.
