# Supplementary material for: Treatment of a Large Cohort of Childhood Chronic Noninfectious Uveitis in a Multicentric Large Study: Adalimumab Versus Methotrexate as First‐Line Therapy
Source: Arthritis Rheumatol. 2026 Mar 12;78(8):1789–97. doi: 10.1002/art.70090 (PMC13430079; doi:10.1002/art.70090)

| * p-values are computed from chi-squared test for categorical variables and from Kruskall-Wallis test for continuous variables |  |  |  |  |  |  |  |
| --- | --- | --- | --- | --- | --- | --- | --- |

Supplementary Table 1:Ocular characteristics at onset and at the last available follow-up and hot they changes across time.

| Variable | Ocular characteristics at onset | Ocular characteristics at the last available follow-up | p value |
| --- | --- | --- | --- |
| Children with complications at onset | 80 (69.0%) | 62 (53.4%) | 0.011 |
| N of complications | 2 (1-4) | 1 (0.0-2.0) | <0.001 |
| BCVA  Logmar  <0.3  0.4-1  >1 | Median  0.2 (0.0-0.5)  49 (42.2%)  21 (18.1%)   1. (9.5%) | 0.0 (0.0-0.15)  91 (78.4%)  18 (15.5%)  6 (5.2%) | <0.001  <0.001 |

**Supplementary Figure 1:** Schematic representation of the decision process for the inclusion of children with cNIU in this study.

SECOND LINE THERAPY

FIRST LINE THERAPY

**Supplementary Figure 2:** Survival curve of time to flare after achieving ocular remission comparing adalimumab when used as first-line treatment and second line treatment (Log-rank, χ²5.28, p0.021).


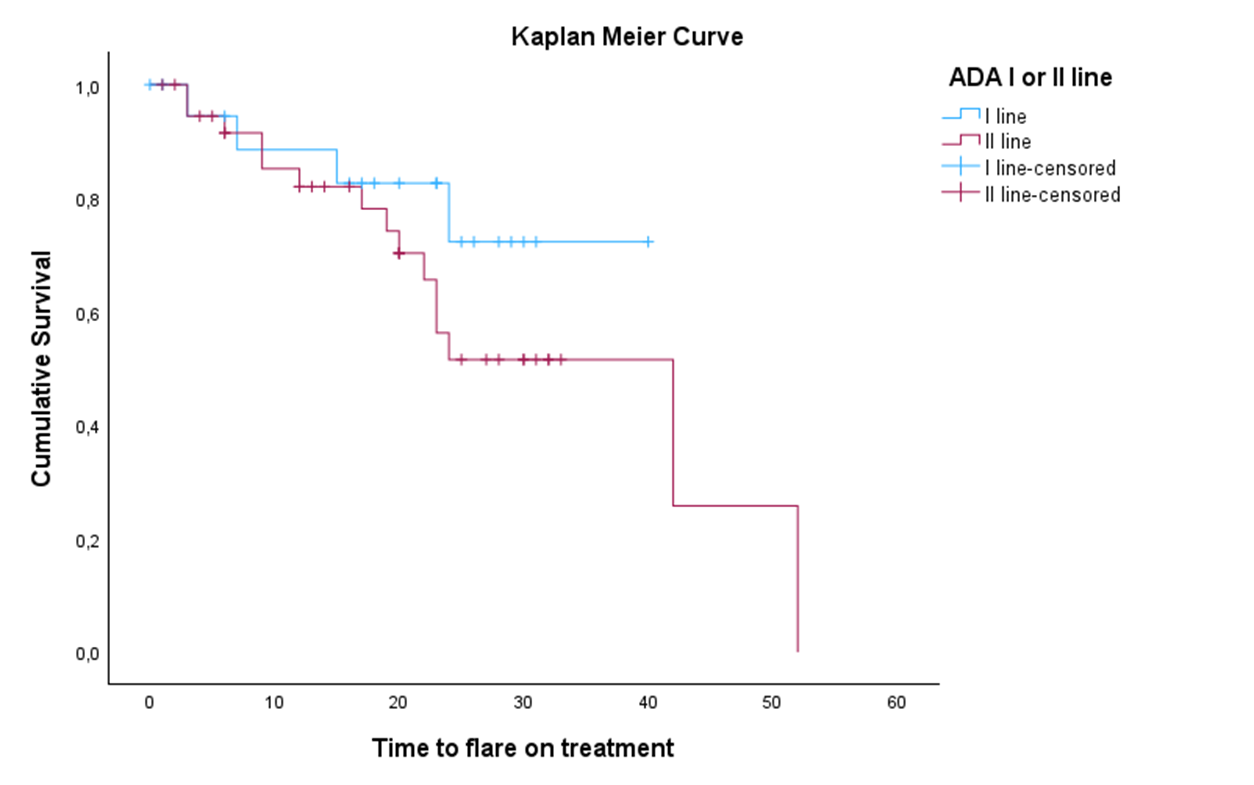

Supplement: Supplementary file 2 — Data S1. Supporting Information. [file ART-78-1789-s002.docx]
